# Xueliankoufuye Suppresses Microglial Activation with Inflammatory Pain by Blocking NF-*κ*B Signaling Pathway

**DOI:** 10.1155/2023/1508098

**Published:** 2023-02-21

**Authors:** Shuai Shao, Yazi Wei, Xingtong Liu, Tiantai Zhang

**Affiliations:** ^1^Institute of Materia Medica, Chinese Academy of Medical Sciences & Peking Union Medical College, Beijing 100050, China; ^2^Shanghai Wenyun Biotechnology Co, Ltd., Shanghai 200030, China

## Abstract

Xuelian, as a traditional Chinese ethnodrug, plays an important role in anti-inflammation, immunoregulation, promoting blood circulation, and other physiological functions. It has been prepared into different traditional Chinese medicine preparations for clinical use, with xuelian koufuye (XL) being widely used to treat rheumatoid arthritis. However, whether XL can relieve inflammatory pain and its analgesic molecular mechanism are still unknown. The present study explored the palliative effect of XL on inflammatory pain and its analgesic molecular mechanism. In complete Freund's adjuvant (CFA)-induced inflammatory joint pain, oral XL dose-dependently improved the mechanical withdrawal threshold of inflammatory pain from an average value of 17.8 g to 26.6 g (*P* < 0.05) and high doses of XL significantly reduced inflammation-induced ankle swelling from an average value of 3.1 cm to 2.3 cm compared to the model group (*P* < 0.05). In addition, in carrageenan-induced inflammatory muscle pain rat models, oral XL dose-dependently improved the mechanical withdrawal threshold of inflammatory pain from an average value of 34.3 g to 40.8 g (*P* < 0.05). The phosphorylated p65 was inhibited in LPS-induced BV-2 microglia and spinal cord of mice in CFA-induced inflammatory joint pain within a value of 75% (*P* < 0.001) and 52% reduction (*P* < 0.05) on average, respectively. In addition, the results showed that XL could effectively inhibit the expression and secretion of IL-6 from an average value of 2.5 ng/ml to 0.5 ng/ml (*P* < 0.001) and TNF-*α* from 3.6 mg/ml to 1.8 ng/ml with IC_50_ value of 20.15 *μ*g/mL and 112 *μ*g/mL respectively, by activating the NF-*κ*B signaling pathway in BV-2 microglia (*P* < 0.001). The above-given results provide a clear understanding of the analgesic activity and mechanism of action not found in XL. Considering the significant effects of XL, it can be evaluated as a novel drug candidate for inflammatory pain, which establishes a new experimental basis for expanding the indications of XL in clinical treatment and suggests a feasible strategy to develop natural analgesic drugs.

## 1. Introduction

Inflammatory pain is a pain response caused by inflammation due to peripheral tissue injury [1]. Nociceptive signals generated after peripheral tissue injury are transmitted to peripheral nerve-ending pain receptors, leading to central sensitization induced by microglia activation [2]. Long-term repeated noxious stimuli maintain the microglia in an activated state, resulting in chronic pain [3, 4]. In response to noxious stimuli, an important molecular event that activates microglia is the activation of the nuclear transcription factor-*κ*B (NF-*κ*B) [5]. It regulates inflammatory signals, responds to extracellular stimulation signals, and induces a large number of proinflammatory cytokines, eventually leading to central sensitization to mediate the upward transmission of inflammatory pain signals [6]. Previous studies confirmed that the expression of proinflammatory factors, including IL-6, TNF-*α*, and IL-1*β* in microglia significantly decreased the mechanical withdrawal threshold of CFA-induced inflammatory pain in rats [7]. In addition, blocking NF-*κ*B activation alleviated microglial activation and allodynia in CFA-induced inflammatory pain [8]. Therefore, inhibition of microglial activation is expected to be a potential strategy for relieving inflammatory pain.

The mechanism of action of analgesic drugs is generally categorized into central analgesia and peripheral analgesia, such as the central analgesic effect produced by the binding of phytocannabinoids to cannabinoid receptors, morphine to opioid receptors, and capsaicin to transient receptor potential channels (TRP channels), and the peripheral analgesia induced by nonsteroidal anti-inflammatory drugs (NSAIDs). However, the long-term use of the above drugs is associated with adverse reactions such as mental addiction or gastrointestinal disorders. Due to the vast diversity and low adverse effects, a series of natural products derived from Chinese herbal medicines have always been the focus and hotspot for drug research and development, especially in managing chronic inflammatory diseases. Ethnic medicine is a treasure of traditional Chinese medicine, with a good safety profile and promising bioactive molecules, which is a strategy to treat inflammatory pain [9, 10]. Bioactive compounds that perform well in controlled clinical trials are also emerging. As a representative of Xinjiang ethnodrug (Uyghur drugs), xuelian has anti-inflammatory and immune regulation and has been made into different traditional Chinese medicines to treat clinical diseases [11]. Xuelian koufuye (XL) is extensively used for treating rheumatoid arthritis and dysmenorrhea caused by insufficient “kidney yang,” “cold dampness,” and “stasis.” However, the possible mechanism by which XL exerts analgesic effects remains unclear; i.e., whether it inhibits pain through anti-inflammatory action must be determined.

In this study, two in vivo inflammatory pain models were established to evaluate the analgesic effect and explore underlying mechanisms. We found that XL, as an anti-inflammatory analgesic, can inhibit the NF-*κ*B signaling pathway to block hyperalgesia caused by excessive activation of microglia.

## 2. Materials and Methods

### 2.1. Drugs and Reagents

XL was provided by Xinjiang Tianshan Lotus Pharmaceutical Co., Ltd. It was dissolved in Dulbecco's Modified Eagle Medium (DMEM) for the experiments. The cell culture reagents and enzyme-linked immunosorbent assay (ELISA) kits were purchased from the Invitrogen Corporation (Thermo Fisher Scientific, Carlsbad, CA, USA). Lipopolysaccharide (LPS), complete Freund's adjuvant (CFA), and carrageenan were acquired from Sigma–Aldrich (St Louis, MO, USA). p-p65, p65, and *β*-tubulin were purchased from Cell Signaling Technology (Beverly, MA, USA). Total glucosides of paeony (TGWP) and diclofenac sodium (DS) were purchased as positive drugs, respectively.

Based on the conversion of data from the adult therapeutic dose to the dose administered to rats, we determined the doses administered for the positive drugs TGWP and DS as 100 mg/kg and 10 mg/kg. The specific conversion process is as follows: the adult daily dose of TGWP and DS is 1.2 g/70 kg and 110 mg/kg, and the rat dose is 6.17 times higher than the adult dose. Therefore, the rat doses of TGWP and DS are approximately 100 mg/kg and 10 mg/kg.

### 2.2. Experimental Animals

Female SD rats (weighing 180–200 g) were obtained from Beijing Huafukang Experimental Animal Institute (Beijing, China). The rats were housed 5 to 6 per cage at room temperature (22 ± 2°C) in specific pathogen-free conditions under 12/12-h reversed light-dark cycles, with food and water provided ad libitum. The rats were acclimatized for 3-4 days prior to the experiments and randomly divided into different groups.

### 2.3. CFA-Induced Inflammatory Joint Pain Model

CFA (100 *μ*L) was injected into each rat's right paw (ipsilateral paw) to construct the model of inflammatory joint pain [12]. The control rats were injected in the same way with normal saline solution. The rats were randomly divided into 7 groups (*n* = 10): control group (sham), model group, three XL-treated groups (250, 500, and 1000 mg/kg), and positive control groups of TGWP (100 mg/kg) and DS (10 mg/kg) treatment. The mechanical withdrawal threshold of the rats in each group was measured at 0.5, 1, 2, and 4 h after daily oral administration.

### 2.4. Carrageenan-Induced Inflammatory Muscle Pain Model

An inflammatory muscle pain model was constructed by injecting 100 *μ*L of 3% carrageenan (pH = 6.0) into the gastrocnemius muscle of the rats [13]. The control rats were injected with normal saline solution in the same way. The grouping, administration, and measurement of the mechanical withdrawal threshold of the rats were the same as the experiments in the CFA-induced inflammatory joint pain model.

### 2.5. Mechanical Withdrawal Threshold of the Paw

The mechanical withdrawal threshold of the rats was measured by the electronic Von Frey withdrawal test (Von Frey filaments, IITC Life Science Inc., California, USA) 30 min, 1 h, 2 h, and 4 h after administration. The animals were acclimatized in boxes set on an elevated metal mesh floor for at least 30 min. The behavioral analyses were performed by an investigator blinded to the experimental grouping.

### 2.6. Cell Culture and Viability Assay

BV-2 microglia were cultured in DMEM supplemented with 10% fetal bovine serum (FBS) at 37°C under 5% CO_2_ and 95% humidity. To assess cell viability, the BV2 cells (2 × 105 cells/mL) were seeded in 96-well plates and incubated at 37°C under 5% CO_2_ with LPS (1 *μ*g/mL) stimulation. After the cells were cotreated with XL with different concentrations and LPS (1 *μ*g/mL) for 24 hours, cell counting kit 8 (CCK8) reagent was added to each well. After 1 hour of incubation, absorbance levels for formazan were measured at 450 nm using a microplate reader (BioTek, VT, USA).

### 2.7. Western Blotting Assay

After preincubation of BV-2 microglia with different concentrations of XL for 1 hour, LPS was used for 24 hours to activate microglia, BV2 cells were washed with phosphate-buffered saline (PBS) solution and treated with 1× cell lysis buffer containing 1× protease and phosphatase inhibitor cocktails. After incubating on ice for 30 minutes, the cells were collected and centrifuged at 12000 rpm for 10 minutes at 4°C. The spinal dorsal lumbar enlargements were separated from mice after measurement of the mechanical withdrawal threshold and then lysed to extract protein. The protein concentration in the cell and tissue lysates were determined by BCA Protein Assay Kit (Thermo Fisher Scientific, Carlsbad, CA, USA), and 20 *μ*g of proteins per sample was separated by 8% sodium dodecyl sulfate-polyacrylamide gel electrophoresis (SDS-PAGE). The bands were transferred to a polyvinylidene fluoride (PVDF) membrane (Millipore Co., Bedford, MA, USA) that was then blocked with 5% bovine serum albumin (BSA) in tris-based saline-Tween 20 (TBST) at room temperature for 1 h. After overnight incubation with the primary antibodies against p-NF-*κ*B p65, NF-*κ*B p65, and *β*-tubulin (all diluted 1 : 1000) at 4°C, the blots were then incubated with goat antirabbit and goat antimouse secondary antibodies. The membrane was developed using enhanced chemiluminescence reagents (PerkinElmer, USA), the bands were visualized with Tanon 2000 Imaging system (Beijing, China), and their intensities were quantified using ImageJ software (NIH, USA).

### 2.8. ELISA Assay

After preincubating BV-2 microglia with different concentrations of XL for 1 hour, microglia were activated with LPS for 24 hours, and then the cell supernatant was collected to detect proinflammatory factors. The concentrations of proinflammatory cytokines in the culture supernatant were determined using an ELISA kit (Invitrogen, Carlsbad, CA, USA) according to the manufacturer's instructions.

### 2.9. Statistical Analysis

The data were expressed as means ± SEM. Statistical significance was determined by two-way ANOVA followed by post hoc Tukey analyses *P* < 0.05 was considered significant.

## 3. Results

### 3.1. XL Alleviated CFA-Induced Inflammatory Joint Pain in Rats

**Figure 1:**
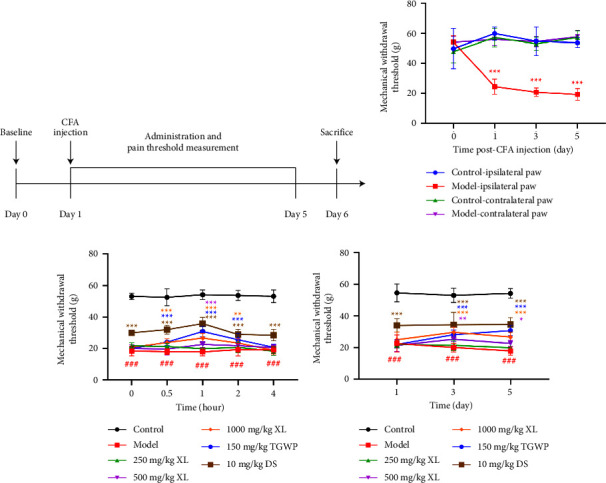
Analgesic effect of XL on complete Freund's adjuvant (CFA)‐induced inflammatory joint pain in rats. The analgesic activity evaluation protocol is operated in accordance with (a). (b) The changes of the mechanical withdrawal threshold induced by CFA were detected by electronic Von Frey test. (c) Paw withdrawal threshold was measured by electronic Von Frey test after oral administration of XL, TGWP, and DS at 30 mins, 1 h, 2 h and 4 h. (d) The analgesic effect at different time points after oral administration XL, TGWP, and DS were detected by electronic Von Frey test. Data are expressed as means ± SEM (*n* = 10 rats in each group). Statistical significance was determined by two-way ANOVA followed by post hoc Tukey analyses where ^*∗∗∗*^*P* < 0.001 vs. control-ipsilateral paw, ^###^*P* < 0.001 vs. control group, ^*∗*^*P* < 0.05, ^*∗∗*^*P* < 0.01, ^*∗∗∗*^*P* < 0.001 vs. model group.

CFA-induced inflammatory joint pain model rats were used to detect the mechanical withdrawal threshold using the electronic Von Frey test to evaluate the effect of XL (Figure 1(a)). Subcutaneous injection of CFA decreased the mechanical withdrawal threshold in the ipsilateral paw from an average value of 53.7 g to 19.2 g, without affecting the contralateral paw (*P* < 0.001; Figure 1(b)). However, 500 mg/kg and 1000 mg/kg of XL significantly increased the mechanical withdrawal threshold in the ipsilateral paw in a dose-dependent manner from an average value of 17.8 g to 26.6 g compared to the placebo-treated mice (*P* < 0.05 or *P* < 0.001; Figure 1(c)). The analgesic effect of XL peaked within one hour, remaining stable over five consecutive days (*P* < 0.05 or *P* < 0.001; Figure 1(d)). Positive control of TGWP and DS did not exhibit a noticeable analgesic effect.

### 3.2. XL Relieved CFA-Induced Paw Thickening and Ankle Swelling

**Figure 2:**
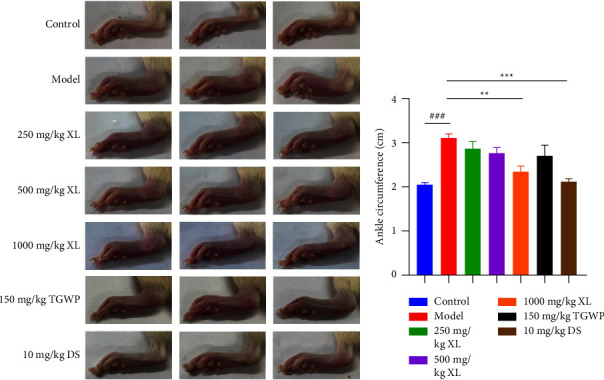
Anti-inflammatory effects of XL on complete Freund's adjuvant (CFA)‐induced inflammatory joint pain in rats. (a) Paw thickness of rats with inflammatory joint pain injected by CFA was shown as microscopic evidences. (b) Ankle circumference was measured in each group of rats. Data are expressed as means ± SEM (*n* = 7 rats in each group). Statistical significance was determined by two-way ANOVA followed by post hoc Tukey analyses where ^###^*P* < 0.001 vs. control group, ^*∗∗*^*P* < 0.01, ^*∗∗∗*^*P* < 0.001 vs. model group.

CFA injection of rat paws induced a severe inflammatory response, with decreased pain threshold and paw swelling. To explore whether XL played an analgesic role through anti-inflammatory action, we observed the degree of paw swelling in each group with continuous daily administration of XL. The results showed that paw thickness was alleviated by the oral administration of XL in the inflammatory joint pain rat model compared to the CFA injection group alone (Figure 2(a)). In addition, ankle circumference was measured in each group of rats. High doses of XL significantly reduced inflammation-induced ankle swelling from an average of 3.1 cm to 2.3 cm compared to the model group (*P* < 0.05; Figure 2(b)).

### 3.3. XL Alleviated Carrageenan-Induced Inflammatory Muscle Pain in Rats

**Figure 3:**
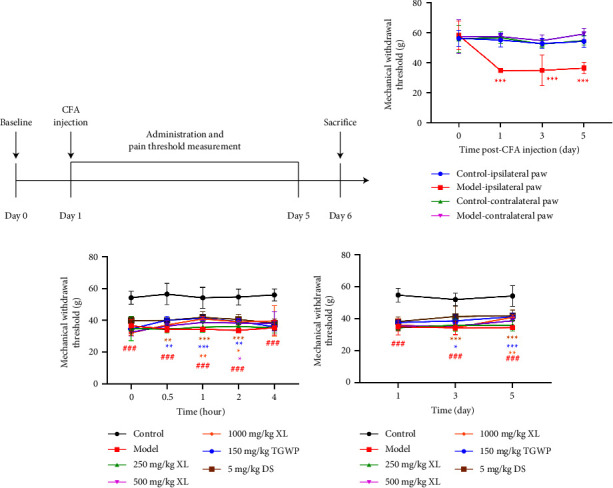
Effect of XL on carrageenan‐induced inflammatory muscle pain in rats. The analgesic activity evaluation protocol is operated in accordance with (a). (b) After carrageenan injection, the mechanical withdrawal threshold of the ipsilateral paw in the model group was significantly reduced, which was statistically different from that of the control group, while the contralateral mechanical withdrawal threshold was not affected. (c) After oral administration of XL 30 mins, 1 h, 2 h and 4 h mechanical withdrawal threshold was measured by electronic Von Frey test. (d) On the first day, the third day, and the fifth day after carrageenan injection, the mechanical withdrawal threshold of rats one hour after oral administration of XL was measured by electronic Von Frey test. Data are expressed as means ± SEM (*n* = 10 rats in each group). Statistical significance was determined by two-way ANOVA followed by post hoc Tukey analyses where ^*∗∗∗*^*P* < 0.001 vs. control-ipsilateral paw, ^###^*P* < 0.001 vs. control group, ^*∗*^*P* < 0.05, ^*∗∗*^*P* < 0.01, ^*∗∗∗*^*P* < 0.001 vs. model group.

To explore the effect of XL on inflammatory muscle pain, we injected 3% carrageenan into the rats' gastrocnemius muscle to induce muscle inflammation (Figure 3(a)). The electronic Von Frey test was used to detect the rats' mechanical withdrawal threshold. Our data showed that carrageenan significantly decreased the mechanical withdrawal threshold in the ipsilateral paw from an average value of 54.2 g to 36.5 g, but not the contralateral paw (*P* < 0.001; Figure 3(b)). A high dose of XL increased the mechanical withdrawal threshold in the ipsilateral paw from an average value of 34.3 g to 40.8 g compared to the placebo-treated mice (*P* < 0.001 or *P* < 0.05; Figure 3(c)). Meanwhile, TGWP and DS also increased the mechanical withdrawal threshold. The analgesic effect of XL peaked within one hour, remaining stable over five consecutive days (*P* < 0.05; Figure 3(d)).

### 3.4. XL Inhibited Painful Response Induced by Microglial Activation via the NF-*κ*B Signaling Pathway

**Figure 4:**
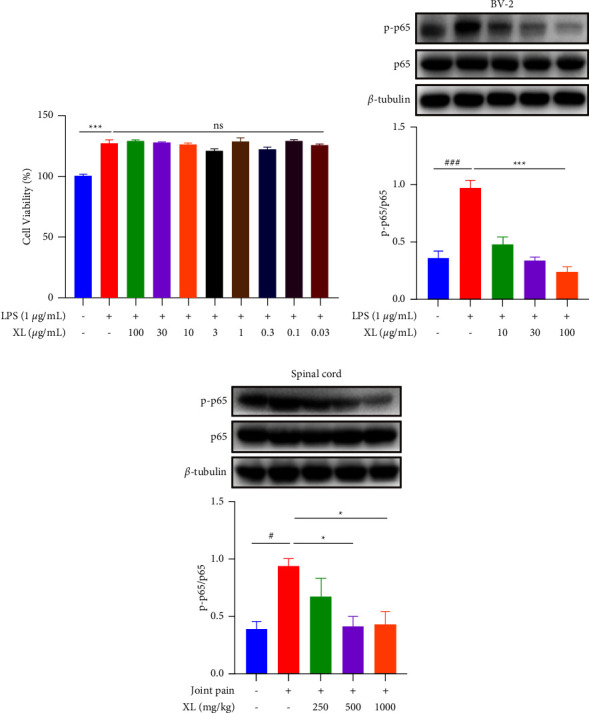
Effect of XL on LPS‐induced the NF-*κ*B signaling pathway in BV-2-microglia and spinal cord. (a) XL did not affect cell viability in BV-2 microglia within LPS stimulation. Cell viability was measured using CCK-8 assay, as indicated in Materials and Methods. (b) At 24 h after LPS treatment, XL inhibited LPS‐induced phosphorylation of the NF-*κ*B p65 in BV2 microglia. (c) Orally administration of XL inhibits p65 phosphorylation of the spinal cord in CFA-induced inflammatory joint pain. Data are expressed as means ± SEM (*n* = 3 in each group). Statistical significance was determined by one-way ANOVA followed by post hoc Tukey analyses where ^*∗∗∗*^*P* < 0.001, ^#^*P* < 0.01, ^###^*P* < 0.001 vs. control group, ^*∗∗∗*^*P* < 0.001 vs. LPS group, ^*∗*^*P* < 0.01 vs. Joint pain group.

Neuroinflammation induced by microglial activation is involved in transmitting inflammatory pain signals. To explore the anti-inflammatory mechanism of XL, we evaluated the effect of XL on LPS-induced NF-*κ*B phosphorylation in BV-2 microglia and the spinal cord. Under LPS stimulation, XL did not affect the viability of BV-2 microglia at a concentration range of 0.03–100 *μ*g/mL (Figure 4(a)). Western blotting results showed that XL inhibited phosphorylation of p65 in BV-2 microglia and spinal cord within the value of 75% and 52% reduction on average, respectively (*P* < 0.001 or *P* < 0.05; Figures 4(b) and 4(c)).

### 3.5. XL Inhibited Secretion of Proinflammatory Cytokines Induced by Microglial Activation via the NF-*κ*B Signaling Pathway

**Figure 5:**
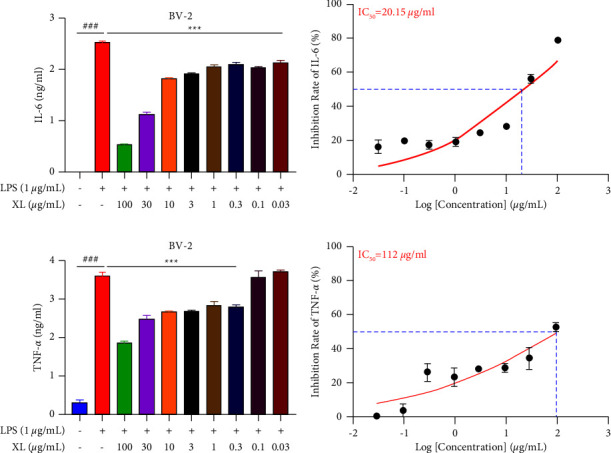
Effect of XL on LPS‐induced secretion of proinflammatory cytokines in BV-2-microglia. XL inhibited LPS-induced secretion of IL-6 (a) with IC_50_ of 20.15 *μ*g/mL (b) and TNF-*α* (c) with IC50 of 112 *μ*g/mL (d) in BV2 microglial cells. The secretion of IL-6 and TNF-*α* were quantified using ELISA kits, as described in Materials and Methods. Data are expressed as means ± SEM (*n* = 3 in each group). Statistical significance was determined by one-way ANOVA followed by Tukey's post hoc analysis where ^###^*P* < 0.001 vs. control group, ^*∗∗∗*^*P* < 0.001 vs. LPS group.

Since XL inhibited painful responses induced by microglial activation via the NF-*κ*B signaling pathway, we finally investigated the effect of XL on the activation of BV-2 microglia and secretion of proinflammatory cytokines. Compared with the LPS stimulation group alone, XL inhibited the secretion of IL-6 from an average value of 2.5 ng/ml to 0.5 ng/ml (*P* < 0.001; Figure 5(a)) and TNF-*α* from an average value of 3.6 mg/ml to 1.8 ng/ml (*P* < 0.001; Figure 5(c)) in the culture supernatant of BV-2 microglia, with IC_50_ value of 20.15 *μ*g/mL (Figure 5(b)) and 112 *μ*g/mL (Figure 5(d)) in a dose-dependent manner, respectively. The above-given results indicated that XL inhibited the neuroinflammation induced by LPS.

## 4. Discussion

The search for therapeutic agents with anti-inflammatory and analgesic effects in natural products has always been an important direction to relieve inflammatory pain, and many active ingredients have been evaluated in a large number of animal pain models in preclinical studies, including inflammatory pain induced by acetic acid, carrageenan, and formalin [14–16]. Although XL is widely used in the clinic for various diseases, its efficacy in relieving inflammatory joint pain remains unclear. Here, our study showed for the first time that XL could effectively relieve inflammatory joint pain induced by CFA and muscle pain induced by carrageenan in a rat model, respectively. This gives us a hint that XL has the potential to become a pharmaceutical formulation for the treatment of inflammatory pain.

Proinflammatory factors play an essential role in inflammatory diseases. Excessive inflammatory responses induce peripheral and central pain sensitization, which is also the main pathway for different types of inflammatory pain [17]. Therefore, natural products with significant anti-inflammatory effects intervene in central and peripheral pain responses by effectively inhibiting nociceptive sensations and inflammatory receptors [18]. Further research indicated that XL inhibited the expression of IL-6 and TNF-*α* in LPS-induced BV-2 cells. Mechanistically, XL suppressed the phosphorylation of p65 of the NF-*κ*B signaling pathway in activated BV-2 cells involved in inflammatory pain by activating the inflammatory signaling. Thus, XL could be a potential therapeutic strategy to alleviate the inflammatory pain associated with microglial activation.

Neuroinflammation induced by microglial activation mediates the pathological development of inflammatory pain [19]. Noxious stimuli acting on microglia can induce microglial activation with a strong phagocytic function and inflammatory response. Therefore, many proinflammatory cytokines are produced, including IL-6, TNF-*α*, and iNOS promoting the inflammatory response in the damaged area and aggravating the damage to neurons, leading to the pain nerve signal transmission and sensitization of central pain [20, 21]. Neuroprotection by inhibiting the neuroinflammatory response caused by the overactivation of MAPKs and NF-*κ*B signaling pathways is an important strategy to address disorders associated with inflammatory and neuropathic pain. In this study, in vitro experiments showed that BV-2 microglia exposed to LPS produced a stronger inflammatory response, which can mimic neuroinflammation induced by microglial activation when inflammatory pain occurs. However, XL dose-dependently inhibited the secretion of IL-6 and TNF-*α* produced by BV-2 microglia due to the inflammatory response. The above-given findings demonstrated that XL inhibited LPS-induced microglia activation and neuroinflammation in BV-2 cells.

In an overly aggressive inflammation-induced pain response, cytokines cause the activation of multiple signaling pathways, resulting in the continuous conduction of pain signals [22]. A series of natural derivatives inhibit the activation of pain signaling pathways by reducing the secretion of cytokines such as IL-6 and TNF-*α*, thus achieving analgesic effects [23, 24]. Whether the significant analgesic effect shown by XL is related to the inhibition of these signaling pathways needs further experimental confirmation. Therefore, we next explored the possible molecular mechanism of XL to inhibit the activation of BV-2 microglia that results in neuroinflammation. An important molecular event in microglial activation is the activation of the NF-*κ*B signaling pathway, which regulates cell proliferation, differentiation, survival, and apoptosis by an inflammatory response and the expression of related inflammatory cytokines [25, 26]. Various bioactive ingredients, such as flavonoids and glycosides, can exert anti-inflammatory, antibacterial, antiviral, immunomodulatory, and other biological effects by inhibiting the NF-*κ*B signaling pathway [24]. In the present experiment, XL inhibited the phosphorylation of the transcription factor NF-*κ*B and the downstream expression of IL-6 and TNF-*α* under LPS stimulation. At the same time, whether it is CFA-induced inflammatory joint pain or carrageenan-induced inflammatory muscle pain model, XL has good analgesic effects. Therefore, the above-given findings indicated that XL inhibited the inflammatory cytokines produced by the activation of the NF-*κ*B signaling pathway in spinal microglia, which may be the main mechanism of its analgesic effects.

Previous studies have reported that CFA can effectively activate microglia in the spinal cord to secrete of inflammatory cytokines, causing mechanical allodynia [27, 28]. This is due to the fact that CFA can cause an intrinsic immune response dominated by pathogen-associated molecular patterns (PAMPs), which leads to a persistent inflammatory environment and microglia activation. It has been suggested that microglial activation plays an important role in inflammatory pain. Inhibition of microglial activation can decrease the mechanical hypersensitivity caused by peripheral inflammation and nerve damage [29]. Proinflammatory cytokines also play an important role in maintaining pain. Intrathecal injection of IL-6, TNF-*α*, and IL-1*β* significantly decreased the pain threshold [30, 31], blocking proinflammatory cytokines via antibodies or eliminating pain behaviors linked to inflammation or neuropathic pain [32–34]. Thus, bioactive compounds with significant anti-inflammatory activity are potential therapeutic agents for relieving inflammatory pain and neuralgia. In the present study, XL inhibited the secretion of proinflammatory factors in microglia, such as IL-6 and TNF-*α*. The results demonstrated that XL inhibited CFA-induced mechanical allodynia by blocking microglial activation and secretion of proinflammatory cytokines. This study provides a new experimental basis for expanding the indications of XL in clinical treatment and suggests a feasible strategy to develop natural analgesic drugs. However, there are still some limitations in this study, firstly, although the inflammatory signaling pathway affected by XL in pain response was found, the exact molecular target of action has not been confirmed and further experimental analysis is needed, and secondly, to expand the prospects of XL in pain clinical treatment, more pain models such as cancer pain and diabetic pain are needed to support its preclinical pharmacological activity evaluation, which is a must for the development of natural analgesic drugs.

## 5. Conclusion

**Figure 6:**
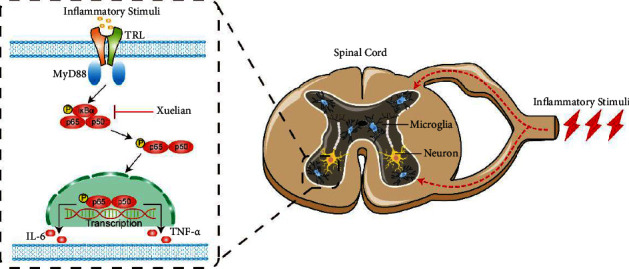
Proposed analgesic mechanism of XL in inflammatory pain.

In summary, inflammatory pain is associated with microglial activation and subsequent NF-*κ*B signaling pathway activation and secretion of inflammatory factors in the spinal cord. In the present study, XL improved the CFA-induced inflammatory joint pain and carrageenan-induced inflammatory muscle pain by raising the mechanical withdrawal threshold of rat paws. Moreover, XL reduces central sensitization in the pain signaling process by inhibiting the expression of inflammatory factors mediated via NF-*κ*B activation in microglia (Figure 6). Taken together, our findings suggest a new indication of XL for expanding the analgesic effect.

## Data Availability

The data used to support the findings of this study are available from the corresponding author upon request.
